# Single and Multiple Change Point Detection in Spike Trains: Comparison of Different CUSUM Methods

**DOI:** 10.3389/fnsys.2016.00051

**Published:** 2016-06-22

**Authors:** Lena Koepcke, Go Ashida, Jutta Kretzberg

**Affiliations:** ^1^Computational Neuroscience, Department of Neuroscience, University of OldenburgOldenburg, Germany; ^2^Cluster of Excellence “Hearing4All”, University of OldenburgOldenburg, Germany

**Keywords:** event detection, spike train analysis, neural coding, signal detection, rate change, moving average, rate coding, response latency

## Abstract

In a natural environment, sensory systems are faced with ever-changing stimuli that can occur, disappear or change their properties at any time. For the animal to react adequately the sensory systems must be able to detect changes in external stimuli based on its neuronal responses. Since the nervous system has no prior knowledge of the stimulus timing, changes in stimulus need to be inferred from the changes in neuronal activity, in particular increase or decrease of the spike rate, its variability, and shifted response latencies. From a mathematical point of view, this problem can be rephrased as detecting changes of statistical properties in a time series. In neuroscience, the CUSUM (cumulative sum) method has been applied to recorded neuronal responses for detecting a single stimulus change. Here, we investigate the applicability of the CUSUM approach for detecting single as well as multiple stimulus changes that induce increases or decreases in neuronal activity. Like the nervous system, our algorithm relies exclusively on previous neuronal population activities, without using knowledge about the timing or number of external stimulus changes. We apply our change point detection methods to experimental data obtained by multi-electrode recordings from turtle retinal ganglion cells, which react to changes in light stimulation with a range of typical neuronal activity patterns. We systematically examine how variations of mathematical assumptions (Poisson, Gaussian, and Gamma distributions) used for the algorithms may affect the detection of an unknown number of stimulus changes in our data and compare these CUSUM methods with the standard Rate Change method. Our results suggest which versions of the CUSUM algorithm could be useful for different types of specific data sets.

## 1. Introduction

It is essential for all animals to properly perceive and interpret their environment, e.g., to avoid predators or to catch prey. Sensory systems transform external signals (e.g., visual or acoustic signals) into corresponding internal representations of neuronal activities, produced by populations of neurons. For the respective sensory system, these spike trains are the only source of information about the environment. In a natural setting sensory systems have to recognize multiple changes in stimulus properties that can occur at any time. Therefore, it is crucial for the sensory systems to detect changes in incoming spike trains caused by changes of relevant external stimuli so that the downstream motor system can generate an appropriate behavior. Delayed or missed perception can lead to dangerous situations or losing prey, whereas false alarms cause loss of energy. Hence, both of them may result in lower survival rates.

**Figure 1:**
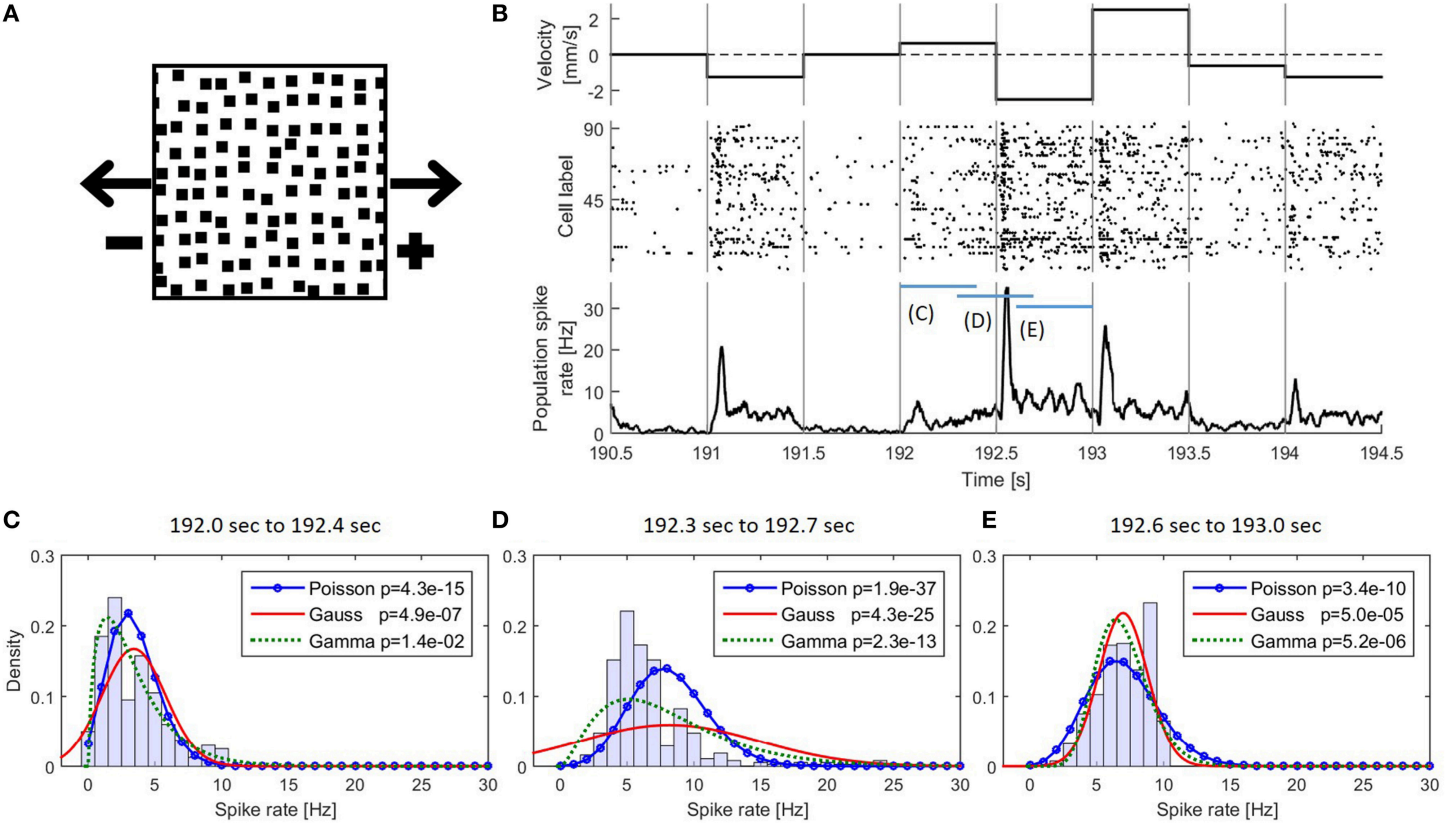
**(A)** Snapshot of the moving light stimulus. **(B)** Example detail of the presented stimulus movement with corresponding responses of the 94 recorded cells. (Top) The dot pattern moved in one of nine different velocities and changed speed and/or direction instantaneously every 500 ms. A positive velocity indicates a movement to the right and negative to the left; (middle) Raster plot of 94 recorded cells; (bottom) PSTH of the pooled activity of all cells smoothed with a rectangular filter of 30 ms bandwidth. **(C–E)** Empirical distributions of the values of the smoothed PSTH in the time intervals **(C)** 192.0–192.4 s **(D)** 192.3–192.7 s **(E)** 192.6–193.0 s together with the three fitted distributions. In the legend *p*-values for testing the respective distributions are shown (see also Methods Section 2.2.1.2).

Neurons can react to different stimulus changes in various ways. Responses vary in spiking activity (e.g., increased or decreased spike rates) and its variability, as well as latencies and spike timings. For example, response latency depends on stimulus intensities in many sensory systems (turtle vision: Greschner et al., 2006; Guillory et al., 2006, salamander vision: Gollisch and Meister, 2010, fly vision: Warzecha and Egelhaaf, 2000, auditory system: Raggio and Schreiner, 1994). Populations of turtle retinal ganglion cells, used as example data in this study, react to stimulus changes with either increased or decreased activity (Thiel et al., 2007). Moreover, neuronal spiking activity is usually not constant over time, even in response to constant stimulation; e.g., adaption can cause large fluctuations (see Figure 1B). Hence, reliable detection of stimulus change despite the large amount of variability of the spike rate is one of the most demanding tasks of the sensory system. And thus, how the nervous systems “decode” spike train data to detect stimulus changes constitutes a central problem of theoretical and computational neuroscience (Rieke et al., 1999; Dayan and Abbott, 2005).

The task to detect changes over time is also relevant in other fields, where statistical properties change in a time series (Basseville, 1988; Gustafsson, 1996; Chen and Gupta, 2000). One standard approach for the analysis of such temporal data is the so-called CUSUM (cumulative sum) method, which was first introduced by Page (1954) based on the earlier work of Hurst (1951). The basic step of this method consists of recursive calculations of calculating a cumulative sum of residuals (Basseville, 1988) and detecting changes of the statistical properties by identifying deviations from a reference state. Because of its simplicity, the idea of CUSUM has been used in a number of applications including neuroscience, where CUSUM has served as an approach to detect single stimulus changes based on the corresponding neuronal responses. Ellaway (1978) first applied the CUSUM procedure to peristimulus time histograms (PSTHs) to uncover small changes in spike rates hidden by random fluctuations. In his work, the values of a PSTH (*y*_*t*_) were simply compared with a reference value ω and the cumulative sum (*S*_*t*_) of the residuals was inspected by eye for detecting changes in the spike rate: $S_{t}=\overset{t}{\underset{i=1}{\sum }}\left(y_{i}-\omega \right)$. Similar methods have been widely used for the analysis of neuroscientific data (Commenges and Seal, 1985; Awiszus et al., 1991; Baker and Gerstein, 2001; Goense and Ratnam, 2003). Davey et al. (1986) analyzed the statistical limits of *S*_*t*_. They introduced a threshold for the cumulative sum to detect a change point under the assumption that the given spike train can be described as a Poisson process. The Ellaway method performs reliably for data sets with small variations (Churchward et al., 1997). In chronic recordings, however, the CUSUM method showed a high probability of missing obvious changes, which required additional parameters to be manipulated continuously for a reliable change point detection (Butler et al., 1992; Churchward et al., 1997).

The CUSUM method can be regarded as hypothesis testing (log-likelihood ratio test), in which the cumulative sum consists of the logarithm of a likelihood ratio (Basseville and Nikiforov, 1993). The likelihood ratio is calculated from two likelihood values with different assumptions: Namely, one with the unchanged condition and the other with changed condition. In a more general framework, Lorden (1971) showed that the CUSUM method is asymptotically optimal if (1) the data is independent and identically distributed and (2) the distributions before and after the change are known. Further studies generalized these results by showing optimality for data that are not necessarily independent and identically distributed (Moustakides, 1986; Ritov, 1990; Moustakides, 1998).

Nevertheless, the CUSUM method requires knowledge about the distributions before and after changes. When it is not possible to estimate empirical distributions before and after the change, like in the case of our example data set, certain parametric distributions have to be assumed. In neuroscience, the Possion, Gaussian and Gamma distributions are most frequently used for analyzing spike data. The underlying process of spiking activity is commonly assumed to be a Poisson process (Davey et al., 1986; Dayan and Abbott, 2005), due to the assumption that the occurrence of a single spike depends solely on time and is not influenced by preceding spikes (see also Supplementary Material 2.1). The Gaussian (or at least a symmetric) distribution is assumed in many analysis techniques used in neuroscience (Churchward et al., 1997; Baker and Gerstein, 2001; Levakova et al., 2015) in which the mean and standard deviation are considered. For the analysis of interspike intervals it is a common approach to assume a Gamma distribution (Baker and Gerstein, 2001; Ratnam et al., 2003). Ratnam et al. (2003), for example, assumed a Gamma distribution for their CUSUM method applied to interspike intervals. Although differences in underlying distributions may affect the performance of the CUSUM method, the validity of these assumptions and their influences on detection performances have rarely been investigated. Using asymptotically optimal CUSUM methods, we thus compare different assumptions on (1) the distribution of the PSTH and (2) the type of shifts in the neuronal activity for the distribution after the change. In particular, we compare the six combinations of (1) Poisson, Gaussian and Gamma distributions and (2) multiplicative or additive shifts as different CUSUM assumptions. We evaluate the methods using published neuronal data from the visual system (Thiel et al., 2007).

The performances of these six CUSUM versions are compared with a simple standard method commonly used in neuroscience, the “Rate Change method” (Baker and Gerstein, 2001; Levakova et al., 2015). It was used, for example, to determine the response latency by calculating the mean and standard deviation from a fixed control period, and identifying response latency when the PSTH exceeded the mean plus/minus a suitable multiple of the standard deviation. This method assumes a symmetric distribution and is similar to the so-called “Xbar and s chart” (Montgomery, 1991).

Previous neuroscientific studies that used the CUSUM method commonly focused on detecting only one stimulus change in the neuronal response. However, in natural situations the sensory system has to detect a sequence of continual stimulus changes of different types. The goal of this study is to introduce change point methods that can detect an unknown number of stimulus changes in a peristimulus time histogram. In our test data unknown and different kinds of stimulus changes trigger a range of neuronal responses which may occur on different time scales. Our goal is to develop an “online” method that bases its decision when a stimulus change occurred only on information available to the nervous systems and can be used for neuronal recordings of any lengths. Hence, the method uses information only on the previous neuronal activity, but not on timing or intensity of the external stimulus. Using a data set from extracellular recordings of the retina that was stimulated with a moving pattern, we here test the above-stated six CUSUM assumptions in comparison to the Rate Change method. First, we divide the experimentally obtained PSTH in segments, each of which contains only one stimulus change, to examine whether these six CUSUM versions lead to different results under the conventional assumption of single stimulus change detection. Next, we use the entire PSTH to test how well the six methods detect unknown numbers of changes. These analyses reveal possible advantages and drawbacks of each of the six CUSUM versions and the Rate Change method.

## 2. Materials and methods

### 2.1. Experimental data

The isolated retina of fresh water turtles (red-eared sliders *Trachemys scripta elegans*) was investigated in electrophysiological experiments with a multi-electrode array. The data used in this study were first published by Thiel et al. (2007) and were re-analyzed here to examine the performance of the CUSUM methods applied to a set of spike data. Animals were used according to the guidelines of the University of Oldenburg Ethics Committee and to ECC rules (86/609/ECC). The retina was stimulated with a pattern of black squares on a bright background moving along the horizontal axis (Figure 1A). The pattern moved in nine different velocities (0, ±0.625, ±1.25, ±1.875, ±2.5 mm/s), where the algebraic sign indicates the movement direction: A positive number refers to movement to the right and a negative number to the left. The number 0 indicates the absence of movement. The velocity remained constant for 500 ms and then changed abruptly (Figure 1B) resulting in 72 (9 × 8) different velocity changes. The stimulus protocol lasted six minutes consisting of ten repetitions of the 72 velocity changes, which were presented in a randomized order. In total, responses to 10 presentations (trials) of this stimulus protocol were recorded. A part of the stimulus protocol is shown in Figure 1B. The activity of the isolated retina was recorded by a 10 × 10 multi-electrode array with a distance of 400 μm between the electrodes. After automatic spike sorting (Offline Sorter, Plexon Inc., Dalles, Texas, USA), recorded responses for every electrode were inspected visually. In this experiment, responses of 94 cells were separated and used for further analysis. Retinal ganglion cell responses to stimulus changes are delayed by signal processing in presynaptic retinal cells (photoreceptors, bipolar, horizontal and amacrine cells). The spike times were adjusted to this minimum latency of 54 ms as described by Thiel et al. (2007).

### 2.2. Change point methods

#### 2.2.1. CUSUM

Here the mathematical background of CUSUM is briefly introduced, for more details see Supplementary Material 2. Let {*y*_1_, …, *y*_*n*_} be data points in a time series where a change in the expected value is assumed. The data points (*y*_*t*_), which are analyzed in this article, are the values of a PSTH, namely the spike rate (or counts) at time *t*. We assume that the data is identically distributed (*y*_*t*_ ~ *f*_μ*t*_) with an expected value μ_*t*_. The mathematical background of the CUSUM approach is hypothesis testing (Basseville, 1988).

(1) $$\begin{array}{l} H_{0}:f_{\mu_{t}}=f_{\mu_{0}},\;\forall t\in \{1,\ldots ,n\}\text{ v.s. }H_{1}:\exists \;c\in \{1,\ldots ,n\}:\\ \;f_{\mu_{t}}=\{\begin{array}{ll} f_{\mu_{0}}, & \forall t\leq c\\ f_{\mu_{1}}, & \forall t>c \end{array}, \end{array}$$

where *H*_0_ is the null hypothesis, *H*_1_ is the alternative hypothesis, *c* is the time of change, μ_0_ is the expected value before the change point *c*, and μ_1_ the expected value afterwards. For testing this hypothesis, the log-likelihood ratio (Fahrmeir et al., 2012) is maximized. The ratio can be calculated recursively:

(2) $$\begin{array}{l} S_{0}=0\text{ and }S_{t}=\max\left\{0,\;S_{t-1}+s_{t}\right\}, \end{array}$$

where the cumulative sum of the residuals (*s*_*t*_) is defined as *s*_*t*_ = ln (*f*_μ1_(*y*_*t*_))−ln (*f*_μ0_(*y*_*t*_)). Here the CUSUM can be interpreted as a cumulative sum of residuals (*s*_*t*_). If *S*_*t*_ from Equation (2) is greater than a threshold α ≥ 0 at a time point *t* = *c*, a change point is detected (Basseville, 1988). When applying the asymptotically optimal CUSUM approach, the following unknowns have to be found:

1. General distribution *f* of the data.
2. Expected value μ_0_ before the change point *c*
3. Type of change of the expected value μ_1_ from μ_0_

We used the following methods to compare different approaches to find these values.

##### 2.2.1.1. CUSUM model assumptions

The empirical distribution of the PSTH changed continuously over time (Figures 1B–E). Even during constant stimulation the empirical distribution of the PSTH was not stable due to different response dynamics of transient and sustained cells in the recorded population. Because of these continuously changing PSTH distribution and the large number of different stimulus changes we used, empirical distributions were not stable enough for change point detection with the CUSUM method.

1. We assumed three different theoretical distributions: Poisson, Gaussian and Gamma distribution. (See next subsection for hypothesis tests comparing these three assumed distributions with empirical distributions).
2. The expected value μ_0_ was estimated from the average value in a predefined interval. The empirical mean is the maximum-likelihood-estimator (Fahrmeir et al., 2012) for the expected value μ.
3. To estimate the relative change of the expected value, we compared two assumptions: a multiplicative (μ_1_ = δμ_0_) and an additive (μ_1_ = μ_0_ + δ) shift of the expected value (see **Tables 2**, **3**).

In total, the six combinations of (1) Poisson, Gaussian or Gamma distribution and (2) multiplicative or additive shift were compared. The residuals *s*_*t*_ in Equation (2) for these assumptions are explained in Tables 1, **3**.

**Table 1:** **Overview of the signs of the residuals *s*_*t*_ of the logarithmic likelihoods (Equation 2) for both hypotheses of increased and decreased activity**.

	**Hypothesis: activity**	
	**increases**	**decreases**
**PSTH values increase**	*s*_*t*_ > 0	*s*_*t*_ < 0
**PSTH values decrease**	*s*_*t*_ < 0	*s*_*t*_ > 0

The spike rate can either increase or decrease in response to a stimulus change (see Figures 1, **3**). Therefore, two different CUSUM test procedures have to be performed in parallel to account for changes in both directions (see Table 2). Equation (2) is valid for both hypotheses (increased and decreased activity), because the residual *s*_*t*_ is always positive if the values of the PSTH change in accordance with the respective hypothesis. Four cases may occur (see Table 1): When the activity increases, the residual is positive (*s*_*t*_ > 0) for the hypothesis of increased activity (μ_0_ < μ_1_) and negative (*s*_*t*_ < 0) for the hypothesis of decreased activity (μ_0_ > μ_1_). Analogously, when the activity decreases, the residual is negative (*s*_*t*_ < 0) for the hypothesis of increased activity (μ_0_ < μ_1_) and positive (*s*_*t*_ > 0) for the hypothesis of decreased activity (μ_0_ > μ_1_). Hence, the residuals *s*_*t*_ defined in Table 3 can be applied to both hypotheses.

**Table 2:** **Overview of all hypothetical changes regarding additive and multiplicative shifts of the expected values μ_0_ and for increased and decreased activity**.

	**Additive shift**	**Multiplicative shift**
**Activity increases**	μ_1_ = μ_0_ + δ_*in*_	μ_1_ = δ_*in*_μ_0_
	(δ*_in_* > 0)	(δ*_in_* > 1)
**Activity decreases**	μ_1_ = μ_0_ + δ*_de_*	μ_1_ = δ*_de_*μ_0_
	(−μ_0_ < δ*_de_* < 0)	(0 < δ*_de_* < 1)

*The additive shift for the decreased activity cannot be less than the expected value μ_0_ because the PSTH has no negative values. Note that for the additive shift δ_de_ has a negative value*.

**Table 3:** **CUSUM formulas for residuals *s*_*t*_**.

	**Additive shift**	**Multiplicative shift**
	(μ_1_ = μ_0_ + δ)	(μ_1_ = δμ_0_)
**Poisson**	$y_{t}\ln\left(\frac{\delta +\mu_{0}}{\mu_{0}}\right)-\delta$	*y*_*t*_ln(δ) + (1 − δ)μ_0_
**Gaussian**	$\frac{\delta }{\sigma^{2}}\left(y_{t}-\mu_{0}-\frac{\delta }{2}\right)$	$\frac{\left(\delta -1\right)\mu_{0}}{\sigma^{2}}\left(y_{t}-\frac{\mu_{0}\left(\delta +1\right)}{2}\right)$
**Gamma**	$k\left(\ln\left(\mu \right)-\ln\left(\mu +\delta \right)+y_{t}\left(\frac{1}{\mu_{0}}-\frac{1}{\mu_{0}+\delta }\right)\right)$	$k\left(-\ln\left(\delta \right)+y_{t}\left(\frac{1}{\mu_{0}}-\frac{1}{\delta \mu_{0}}\right)\right)$

*In this table, s_t_ of Equation (2) is shown for the six different models, for more details see Supplementary Material 2. The parameter space of the relative shift δ is shown in Table 2. Additional distribution parameters are σ^2^ (variance) for the Gaussian methods and k (shape parameter) for the Gamma methods. These equations apply to both increased and decreased activity changes. For the additive shift positive δ indicate increased and negative δ decreased activities. For the multiplicative shift δ > 1 correspond to increased and δ < 1 to decreased activities, respectively (see Table 2)*.

##### 2.2.1.2. Hypothesis tests of the distributions

The empirically determined distribution of the PSTH was not used as CUSUM assumption, because different stimuli elicited different distributions and the PSTH was not stationary (Figures 1B–E). Therefore, we applied hypothesis tests (Kolmogorov-Smirnov-Test: kstest in MATLAB) comparing all three assumed theoretical distributions (Poisson, Gaussian, Gamma) to empirical PSTH distributions. The CUSUM methods were implemented with smoothed PSTHs of different bandwidths (1, 5, 10, 20,…,70 ms) (see e.g., Section 2.3, Table 4 and Supplementary Material 1), and testing was performed for each of these different smoothed PSTHs. For each of the total 7280 stimulus changes (728 changes in each of the ten trials) we tested the distributions of the PSTH values in different response phases within the 500 ms, i.e., (1) transient response phase: 0–100 ms after stimulus change; (2) transient to stable response phase: 100–200 ms after stimulus change; (3) stable response phases (a) 200–400 ms after stimulus change (see Reference Window, e.g., Table 4) and (b) 200–500 ms. For each of these phases, hypothesis tests were performed independently of each other for each bandwidth and for each assumed distribution. Additionally, the PSTHs were pooled according to the seven clusters of population responses (see Section 3.1) and the same tests were performed with these pooled PSTH data. Using a significance level of 0.05, we did not find any evidence that the values of the smoothed PSTHs were Poisson, Gaussian or Gamma distributed.

**Table 4:** **All parameters for the (A) single and (B) multiple stimulus change detection procedure**.

**Parameter**	**Parameter space**	**Poisson Add**	**Poisson Mult**	**Gaussian Add**	**Gaussian Mult**	**Gamma Add**	**Gamma Mult**	**Rate Change**
**A. Single Stimulus Change Detection**								
PSTH bandwidth Δ	{1, 5, …, 70} ms	1	1	5	1	5	5	40
Relative shift δ*_in_*	see Table 2	10	1.1	5.5	1.1	8	1.5	
Relative shift δ*_de_*	see Table 2	−1.75	0.6	−1	0.45	−2	0.5	
Threshold α*_in_*	α*_in_* > 0	10.5	2.2	66	2.1	7.5	6	4.5
Threshold α*_de_*	α*_de_* > 0	5.5	6	15	5.5	8	8.7	3
Reference window *R*	{25, 50, …, 200} ms	200	200	200	200	200	200	200
Bin size	1 ms							
Starting point *t*_*s*_	−100 ms							
**B. Multiple Stimulus Change Detection**								
PSTH bandwidth Δ	{1, 5, …, 70} ms	1	10	40	20	20	20	40
Relative shift δ*_in_*	see Table 2	10	3	6	1.7	18	4.7	
Relative shift δ*_de_*	see Table 2	−3	0.2	−3	0.5	−1	0.6	
Threshold α*_in_*	α*_in_* > 0	10.5	9.5	44	94	16	36	3.2
Threshold α*_de_*	α*_de_* > 0	8.5	8	39	44.5	3.5	78	2
Reference window *R*	{50, 100, …, 500} ms	200	300	400	400	400	400	450
Analysis window *A*	{5, 10, …, 100} ms	50	50	25	45	50	50	
Bin size	1 ms							
Event latency Δ*e*	50 ms							

*The parameter optimization is described in Section 2.4 and Supplementary Material 3. The results for these parameter combinations are shown in Figures 4, 5. The thresholds α_in_, α_de_ were determined separately for each of the ten trials, but did not differ greatly. α_de_ and α_in_ have no unit. The table shows mean values for each method. The parameters not changed systematically (bin size, starting point t_s_ and event latency Δe shown in the bottom of the tables) were used for all methods. For an easier comparison the additive models are shaded in gray. The relatives shifts δ have the unit spikes/s for the additive models, whereas for the multiplicative models δ have no unit*.

#### 2.2.2. Rate change method

In contrast to the CUSUM methods, in which the temporal sum of the residuals was used to detect changes, the Rate Change method directly evaluates the data value at each time point. The mean ${\bar{y}}_{t}$_*t*_ and the standard deviation *sd*_*t*_ was determined in a certain time window of length *R*. A change point was identified when the data point *y*_*t*_ deviated from the mean by a predefined number (α_*in*_,α_*de*_) of standard deviations (Levakova et al., 2015):

(3) $$\begin{array}{l} y_{t}>{\bar{y}}_{t}+\alpha_{in}sd_{t}\text{ or }y_{t}<{\bar{y}}_{t}-\alpha_{de}sd_{t} \end{array}$$

This method implicitly assumes that the underlying distribution in the reference window is symmetrical, even though different factors α_*in*_ for increasing and α_*de*_ for decreasing activities were applied.

### 2.3. Implementation

The CUSUM approach and the Rate Change method were used in previous studies to detect only one stimulus change from neuronal responses (Ellaway, 1978; Awiszus et al., 1991; Levakova et al., 2015). In a natural situation, however, the nervous system has to identify an unknown number of stimulus changes of various types. All methods were applied to detect both a single change and a sequence of an unknown number of stimulus changes. An “event” is defined as a distinct change in the response properties. A “correct event” is a detected change in response to a stimulus change, while a “false event” is defined as a detected change of the response properties without a stimulus change being present. In our analysis, an event was regarded as correctly detected if the detection occurred within the interval 5 ms before and 90 ms after a stimulus change (plus the latency adjustment of 54 ms: see Section 2.1). This time range accounts for the distribution of response latencies we found for different stimuli. This number of 90+54 ms response latency corresponds to the value reported by Thorpe et al. (1996), who found that the human visual system needs about 150 ms after stimulus onset to process a complex natural image.

#### 2.3.1. Single stimulus change detection

**Figure 2:**
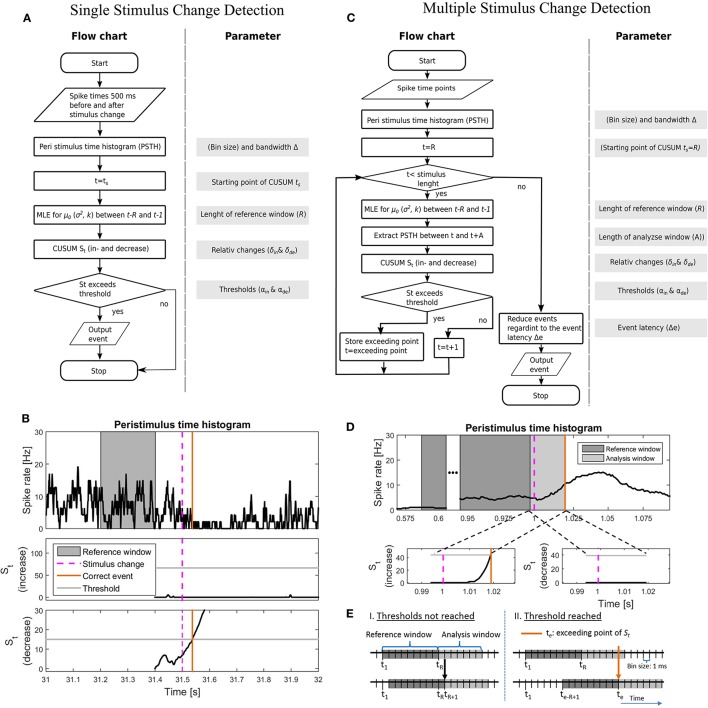
**Illustration of the implementations of the CUSUM approach for the single (A,B) and multiple (C–E) stimulus change detection procedures**. **(A)** Flowchart and a list of parameters for the single stimulus change detection. The parameters are listed in Table 4A. **(B)** An example of the implementation for single stimulus change detection on the example for the Gaussian additive model. Values of the parameters are shown in Table 4A. (Top) PSTH with a reference window, stimulus change and correct detected event; (middle) Cumulative sum *S*_*t*_ for the increased activity with threshold α_*in*_; (bottom) Cumulative sum *S*_*t*_ for the decreased activity with threshold α_*de*_. Both cumulative sums were calculated in parallel. **(C)** Flowchart and included parameters for the single stimulus change detection. The parameters are listed in Table 4B. In **(A,C)**, MLE stands for maximum likelihood estimation. **(D)** An example of the implementation for multiple stimulus change detection on the example for the Gaussian additive model. The parameter values are shown in Table 4B; (Top) PSTH with reference window, analysis window, stimulus change and correct detected event; (bottom-left) Cumulative sum *S*_*t*_ for the increased activity with threshold α_*in*_; (bottom-right) Cumulative sum *S*_*t*_ for the decreased activity with threshold α_*de*_. Both cumulative sums were calculated in parallel. PSTH for the multiple change detection **(D)** was smoother than that for the single change detection **(B)**, because of the longer PSTH bandwidth Δ after optimization (single: 5 ms; multiple: 40 ms, see Table 4). **(E)** Visualization of how to shift the reference window. I. if the cumulative sum *S*_*t*_ did not reach any threshold (α_*in*_, α_*de*_) or II. if a threshold was reached.

In many applications of change point detection, a predefined interval prior to the stimulus change was used as reference data set (Ellaway, 1978; Churchward et al., 1997), to test for changes in response. This approach requires prior knowledge about the timing of stimulus changes. When analyzing single stimulus changes, we assumed that every stimulus change was independent of the others (see Figure 2). Therefore, the PSTH was split into intervals of 800 ms, containing the data of 300 ms before and 500 ms after each stimulus change. To exclude the influence of the previous stimulus change, only the last 300 ms of the response to the previously present constant velocity stimulation were used for the analysis of the current stimulus change.

##### 2.3.1.1. CUSUM

Table 4A summarizes all parameters optimized for the CUSUM methods. The bin size of the PSTH was set to 1 ms. Let {*y*_−299_, …, *y*_0_, *y*_1_, …*y*_500_} be the values of a smoothed PSTH (see Supplementary Material 1), where a stimulus change happened at time point 1. Several parameters (e.g., $\mu_{0},\sigma^{2},k$; see Table 3) had to be determined for each of the CUSUM versions. A predefined reference interval ([*t*_*s*_ − *R, t*_*s*_ − 1]) of length *R* > 0 before the stimulus change was needed to calculate the maximum likelihood estimators (${\hat{\mu }}_{0},{\hat{\sigma }}^{2},\hat{k}$). *t*_*s*_ < 0 is the starting time point of CUSUM (*S*_*t*_). *t*_*s*_ was not optimized by parameter search because of the trivial relationship that the closer the time *t*_*s*_ is located to the stimulus change, the higher must be the detection performance. Within the interval 5 before to 90 ms after the stimulation change an event was considered as correctly detected. To be sure that an event is correctly detected, the starting point of CUSUM was set to 100 ms before the stimulus change (*t*_*s*_ = −100), so the cumulative sum should not reach any threshold in the same magnitude of time length. The reference window was chosen in a way that it was located in a constant stimulation condition without any influence of the previous stimulus. Therefore, *R* was restricted to a maximum length of 200 ms (see Table 4A). The methods should be reliable also under constant stimulation, where no changes should be detected. The equations for the maximum likelihood estimators are summarized in the Supplementary Material 2. Because of the two cumulative sums (for increased and decreased activities) computed at the same time, only the one that first reached the threshold was considered. The flow chart and a sketch of this procedure are shown in Figures 2A,B.

##### 2.3.1.2. Rate change method

As for the CUSUM methods the starting point *t*_*s*_ of the Rate Change method was set to 100 ms before the stimulus change. The mean and standard deviation were estimated in the reference window [*t*_*s*_ − *R, t*_*s*_ − 1] of size *R* (≤ 200 ms). Every data point *y*_*t*_ was compared to the mean and standard deviation obtained for the reference window as described in Equation (3).

#### 2.3.2. Multiple stimulus change detection

##### 2.3.2.1. CUSUM

In contrast to the prior procedure, where only one single stimulus change was considered at a time, real data sets may contain an unknown number of stimulus changes (see Figures 1B, 2C,D). The approach for detecting multiple stimulus changes is similar to the procedure for detecting a single change. The maximum likelihood estimators were calculated from the smoothed PSTH in a reference window of length *R* (see Supplementary Material 1). Therefore, the first possible starting point of the cumulative sum was time point *R*. In addition to the previous procedure, a maximum analysis length (*A*) of the cumulative sum *S*_*t*_ needed to be determined. This time window of the length *A* is called the analysis window. If any threshold was reached within this analysis window the next starting point of CUSUM was set to the point where *S*_*t*_ exceeded the threshold. Otherwise the starting point was shifted by one time step (Figure 2E). Here, we introduced an event latency (Δ*e*) during which no additional events could be detected. Δ*e* was set to 50 ms to account for the high values in the auto-correlation of the PSTH within this range. In mathematical terms, this means:

(4) $$E_{0}(t)=\{\begin{array}{ll} 1, & \text{ if }S_{t}>\alpha \\ 0, & \text{otherwise} \end{array},\;t\geq R$$

(5) $$\begin{array}{ccc} \text{Events} & = & \left\{t\;|\;E_{0}\left(t\right)=1\wedge E_{0}\left(t^{*}\right)=0\;\forall \;t-\Delta e\leq t^{*}<t\;\right\} \end{array}$$

##### 2.3.2.2. Rate change method

In the Rate Change method for detecting multiple stimulus changes the reference window was shifted at every time point by one time bin. The first possible starting point for this method was also time point *R*. For every time point *t* the mean and standard deviation were estimated in the interval [*t* − *R, t* − 1] and then compared with the data point *y*_*t*_ (see Equation 3). The same event latency as for the CUSUM methods was required (Δ*e* = 50) and used as in Equation (4) and (5).

### 2.4. Parameter optimization

In the application of a CUSUM method, a large number of parameters have to be optimized. Because the number of possible parameter combinations is virtually infinite, not all parameter combinations could be tested. For every trial, the thresholds of one parameter combination were optimized using the experimental data of nine trials out of ten. At each optimization step, one trial was excluded to avoid overfitting (leave-one-out cross validation). The parameters were optimized by an optimization function *P* which combines the weighted values of correct (*E*_*true*_) and false (*E*_*false*_) events.

(6) $$\begin{array}{ccc} E_{true} & = & \frac{\text{Number of correct events}}{\text{Number of stimulus changes}},\\ E_{false} & = & \frac{\text{Number of false events}}{\text{Number of stimulus changes}} \end{array}$$

Because we consider a high percentage of detected stimulus changes to be more important than a low number of false events, the total performance *P* was defined as

(7) $$\begin{array}{l} P=2E_{true}-E_{false}. \end{array}$$

The optimal parameter combinations are shown in Table 4A (single stimulus change) and Table 4B (multiple stimulus changes). The parameter optimization strategy is briefly described in Supplementary Material 3. The Rate Change method has a lower number of parameters to be optimized than the CUSUM methods. Namely, the bandwidth Δ, reference window *R* and the multiples of standard deviation (α_*in*_, α_*de*_) were adjusted with the same procedure as for the CUSUM methods.

### 2.5. Evaluation of the results

For the analysis of the results several additional measures were calculated based on *E*_*true*_, *E*_*false*_ and *P* (see Equations 6 and 7).

#### 2.5.1. Single stimulus change detection

For this procedure *E*_*no*_, *E*_*early*_ and *E*_*late*_ were calculated. *E*_*no*_ is the fraction of stimulus changes for which the cumulative sum *S*_*t*_ did not cross any of the thresholds (α_*in*_, α_*de*_). Hence, *E*_*no*_ = 1 − *E*_*true*_ − *E*_*false*_. The false events can be separated into events detected too early (*E*_*early*_) and events detected too late (*E*_*late*_). Events detected too early were detected in the interval between the starting point *t*_*s*_ and the velocity change. Accordingly, a false event was an event detected too late, if the detection occurred later than 90 ms after the velocity change. Therefore, *E*_*false*_ = *E*_*early*_ + *E*_*late*_.

#### 2.5.2. Multiple stimulus change detection

For the detection of multiple stimulus changes, it is not possible to determine unambiguously if a false event is detected too early or too late. Hence, the relative frequencies *E*_*missed*_, *E*_*double*_ and *E*_*stoch*_ were calculated instead. *E*_*missed*_ = 1 − *E*_*true*_ describes how often stimulus changes were missed. The false events were separated into repeated detection of a stimulus change *E*_*double*_ and stochastic events *E*_*stoch*_. The double events (*E*_*double*_) describe the fraction of stimulus changes which were detected twice, meaning that a second event was identified within 90 ms after the stimulation change. The stochastic events *E*_*stoch*_ originate from fluctuations of the spike rate during constant stimulation. The relation between these events was *E*_*false*_ = *E*_*double*_ + *E*_*stoch*_. Note that the sum of the correct, false and missed events was not equal to 1 because more than one event could be detected in the same 500 ms period of constant stimulation (*E*_*true*_ + *E*_*missed*_ + *E*_*false*_ ≥ 1).

### 2.6. Effect of population size

To examine the effect of the population size on the resulting performances, we applied all change points methods to down-sized data sets (**Figure 6**). We show the results for data sets with {1,5,10,…,50, 60,…,90} different cells, randomly taken from the total population consisting of 94 cells. For every population size at least ten different data sets were used for the analysis. Additionally we ensured that every cell was chosen at least once in one of these data sets. Hence, 94 data sets of one cell, 19 of five cells and 10 data sets of each population size between 10 and 90 cells were analyzed.

## 3. Results

The comparison of the different methods presented here is based on a multi-electrode recording of 94 turtle retinal ganglion cells responding to moving light stimuli (see Section 2.1, and Figure 1).

### 3.1. Neuronal responses to stimulus changes

**Figure 3:**
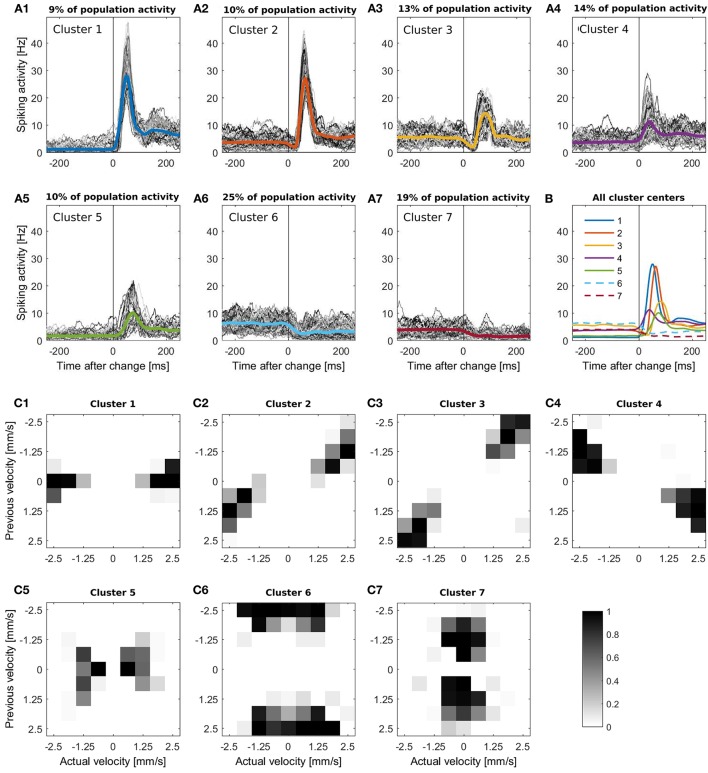
**(A1-A7)** K-means clustering of PSTHs into seven clusters. The PSTH was smoothed with a bandwidth of 30 ms and segmented in 500 ms periods, containing the intervals of 250 ms before to 250 ms after each stimulus change. **(A1-A7)** All responses of each cluster, the respective colored line illustrates the cluster mean. The clusters contain **(A1)** 639 **(A2)** 759, **(A3)** 936, **(A4)** 1008, **(A5)** 731, **(A6)** 1836 and **(A7)** 1381 stimulus changes; **(B)** Comparison of all seven cluster centers. Clusters with activity decreases are represented by a dashed line; **(C1-C7)** Distribution of the corresponding stimulus changes for every cluster. The x-axis represents the actual velocity present after the stimulus change, y-axis the previous velocity. The gray scale of these figures indicates the percentage of the responses to each stimulus change (from a specific previous to a specific actual velocity) included in the cluster.

In Figure 1B the responses of the 94 cells are illustrated in a raster plot and in a smoothed peristimulus time histogram (PSTH; see Supplementary Material 1). Even during presentation of a stimulus moving with a constant velocity, the recorded population-average spike rate showed considerable variability. Changes in stimulus velocity led to changes in spike rate, which depended on the combination of velocities before and after the change either increased or decreased. For visualization and interpretation of the results all responses were categorized into seven clusters (see Figures 3A1–A7) to generate a lower dimensional data set than the original 72 different velocity changes. The PSTH in each time interval of 250 ms before and after a stimulus change were categorized with K-means clustering (MATLAB function *kmeans*) using the Euclidean distance (Xu and Wunsch, 2005). By this clustering procedure typical response patterns of PSTHs (increases of different amplitudes and latencies (Figures 3A1,A4,A5), biphasic activity changes (Figures 3A2,A3), and decreases of two different dynamics (Figures 3A6,A7) were obtained, which could also be found similarly in different neuronal systems. The number of clusters was chosen to yield at least two clusters each with increased and with decreased activities, and seven was the lowest number satisfying this requirement. Note that parameter optimization of the CUSUM method was based on the original data set, not the clusters.

Figures 3C1–C7 shows for each of the seven clusters how often the clustered responses were elicited by each of the stimulus changes from previous to actual stimulus velocity. We found that the seven clusters of similar response time courses corresponded to different categories of velocity changes.

1. Cluster 1 (Figure 3A1) contained strong, transient peaks in population activity, occurring with a short latency after stimulus changes. After the peak, steady state activity was considerably higher than before. These responses were mainly triggered by stimulus changes from no movement (previous velocity 0) to stimuli moving at a high speed in either direction (actual velocity ±2.5 or ±1.875 (Figure 3C)).
2. Responses in cluster 2 (Figure 3A2) also displayed steady state increases and spike count peaks of similar amplitudes as in cluster 1, but with a longer latency due to a preceeding short dip in spike count. These responses were caused mainly by stimuli switching direction and increasing speed (Figure 3C2).
3. The biphasic nature of responses is even more pronounced in cluster 3 (Figure 3A3), featuring a longer, deeper dip followed by a smaller peak in population activity and no difference in steady state response before and after the stimulus change. This response type corresponded to high speed movements reversing direction without changing speed (Figure 3C3).
4. In responses of cluster 4 (Figure 3A4), a small peak in spike rate occurred after a very short latency, followed by an increase in steady state activity. This cluster contained responses to speed increases from a low-to-medium to a high value in the same direction (Figure 3C4).
5. Responses in cluster 5 (Figure 3A5) were characterized by a low steady state activity before the stimulus change and a small activity peak occurring after a long latency. These sluggish activity increases were triggered by low stimulus speeds which either increased slightly or reversed direction (Figure 3C5).
6. The last two clusters contained the traces with reduced activity. Responses in cluster 6 (Figure 3A6) started with a high baseline activity which decreased due to a change from a high initial speed followed to a lower speed regardless of a change in direction (Figure 3C6).
7. In contrast, the initial baseline activity in cluster 7 (Figure 3A7) was low, due to a low/medium initial stimulus speed (Figure 3C7) and was further reduced by a stimulus change to a lower speed in either direction.

For all seven clusters the corresponding distributions of the velocity changes were point-symmetric, indicating that the two directions of stimulus movement could not be distinguished on the basis of the pooled population activity.

### 3.2. Change-point analysis

In this section the results for the single and the multiple stimulus change detection procedures are described. For the single stimulus change detection responses to every stimulus change were analyzed independently from the other changes, whereas for the multiple case one PSTH with a sequence of an unknown number of stimulus changes was examined.

#### 3.2.1. Single stimulus changes

Figure 2B shows an example of detecting a single stimulus change for the Gaussian additive model. The smoothed PSTH and both cumulative sums (for increased and decreased activity) are illustrated. There, the stimulus change induced an activity decrease that was detected about 40 ms after stimulus change. At this time point the cumulative sum *S*_*t*_ for the activity decrease hypothesis got enough evidence to detect an event.

**Figure 4:**
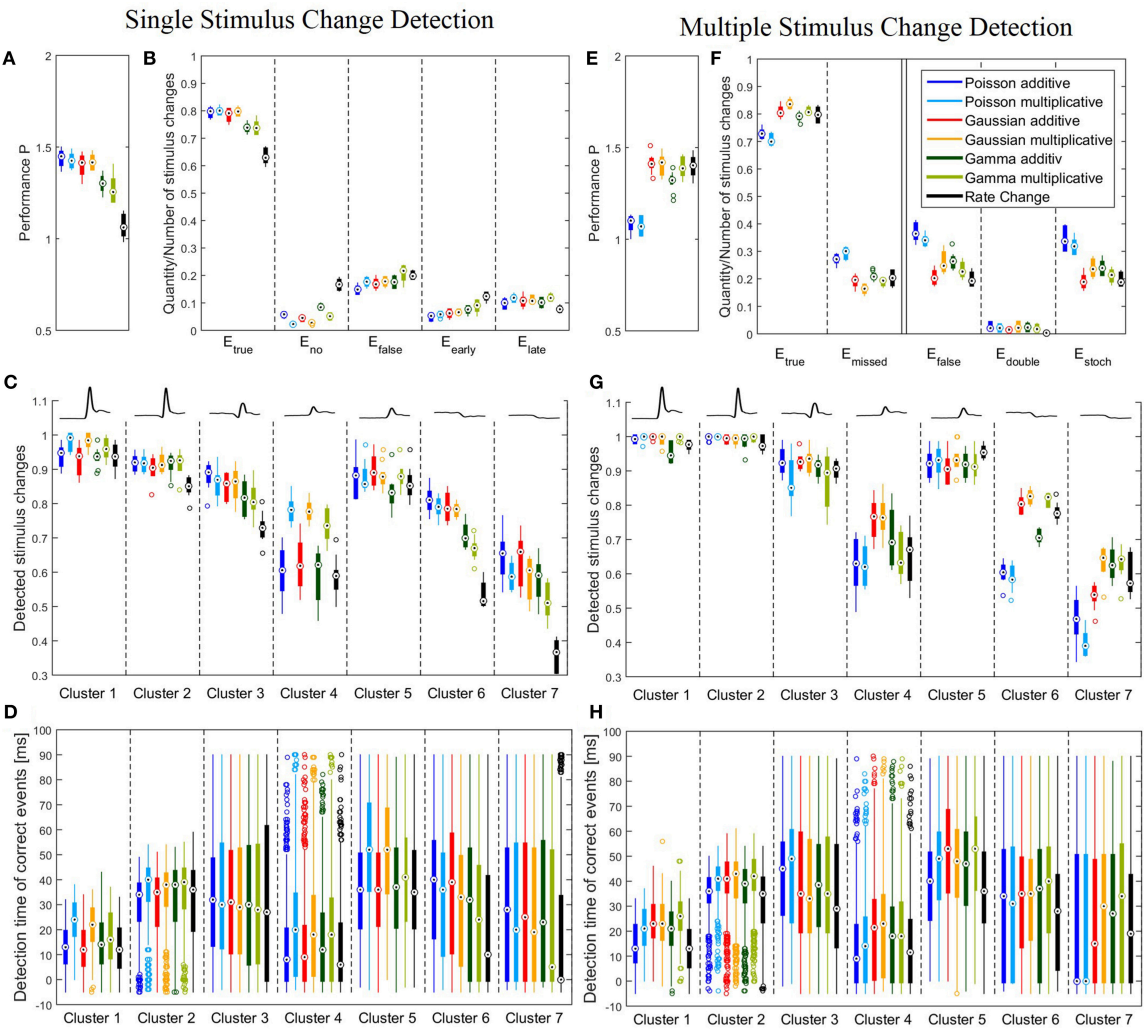
**Performance of all six CUSUM versions and the Rate Change method**. **(A–D)** Single stimulus change detection with the optimized parameters listed in Table 4A. **(E–H)** Multiple stimulus change detection with the optimized parameters listed in Table 4B. Boxplots **(A–C,E–G)** show distribution of performances obtained for the 10 iterations of the stimulus protocol. For each stimulus trial, the performance was calculated by the percentages of correct and false events for all 728 stimulus changes **(A,B,E,F)** or all stimulus changes included in each of the clusters, respectively **(C,G)**. Boxplots **(D,H)** display distributions of all individual detection times obtained in all 10 stimulus iterations (see legend of Figure 3 for number of stimulus changes). **(A)** Boxplots of the total performances *P* of Equation (7); **(B)** Relative frequencies of the correct (*E*_*true*_), missed (*E*_*no*_), false (*E*_*false*_), too early (*E*_*early*_) and too late (*E*_*late*_) events. *E*_*true*_ and *E*_*false*_ were calculated from Equation (6) and *E*_*no*_, *E*_*early*_ and *E*_*late*_ were determined as described in Section 3.2.1. Note that *E*_*true*_ + *E*_*no*_ + *E*_*false*_ = 1 and *E*_*early*_ + *E*_*late*_ = *E*_*false*_. **(C)** Boxplots of the correct events for the different response clusters. The mean cluster PSTHs are illustrated in the top. **(D)** Boxplots of the detection time of the change points for the correct events relative to the stimulus change for each cluster. Note that the allowed range of detection times was between -5 and 90 ms because of the latency adjustment (see Sections 2.1 and 2.3). **(E)** Boxplots of the total performances *P* of Equation (7) for multiple stimulus change detection, **(F)** Relative frequencies of the correct *E*_*true*_, missed (*E*_*missed*_), false (*E*_*false*_), double (*E*_*double*_) and stochastic (*E*_*stoch*_) events. *E*_*true*_ and *E*_*false*_ were calculated from Equation (6) and *E*_*missed*_, *E*_*double*_ and *E*_*stoch*_ were determined as described in Section 3.2.2. Note that *E*_*true*_ + *E*_*missed*_ = 1 and *E*_*double*_ + *E*_*stoch*_ = *E*_*false*_ **(G)** Boxplots of the correct events for the different response clusters. The mean cluster PSTHs are illustrated in the top. **(H)** Boxplots of the detection time of the correct events for each cluster.

The parameters for the single stimulus change detection were chosen to maximize the total performance *P* (see Equation 7). The optimization strategy is described in Section 2.4 and Supplementary Material 3. The optimized parameters for all six methods are summarized in Table 4A. All CUSUM models required a small bandwidth Δ and the maximum length of reference window *R* considering that the reference window had to be located in constant stimulation conditions. The Rate Change method required the same reference window and a considerably longer bandwidth Δ than the CUSUM methods. The performances for all seven methods are shown in Figures 4A–C. Figure 4A illustrates the total performance *P* = 2*E*_*true*_ − *E*_*false*_ (Equation 7) resulting from the parameter optimization. Figure 4B represents the percentage of stimulus changes leading to correct detection of events (*E*_*true*_), no detection (*E*_*no*_) and detection at incorrect times (*E*_*false*_). False detection occurred too early (*E*_*early*_) or too late (*E*_*late*_) for corresponding to the stimulus change (see Section 2.5.1 for the definitions).

Figures 4A,B show that single stimulus changes can be detected reliably with all six CUSUM methods. Both Poisson and Gaussian models detected about 80% of the stimulus changes, while yielding approximately 15% of false detections for both additive and multiplicative approaches. Therefore, these four models achieved similar total performances *P*. Both additive and multiplicative Gamma models yielded sightly lower performances than the Poisson and Gaussian models. The amount of correctly detected events was about 5% lower (ca. 75%). Looking at differences between additive and multiplicative assumptions for all three distributions the frequency of false detections was higher for the multiplicative case. On the other hand, the additive versions show a higher tendency for detecting no events because the cumulative sum (*S*_*t*_) did not cross any thresholds. In contrast to the CUSUM methods the Rate Change method achieved lower scores. Only about 60% of the stimulus changes were detected in the accepted time window. The change points were more likely to be missed or detected too early (Figure 4B).

**Figure 5:**
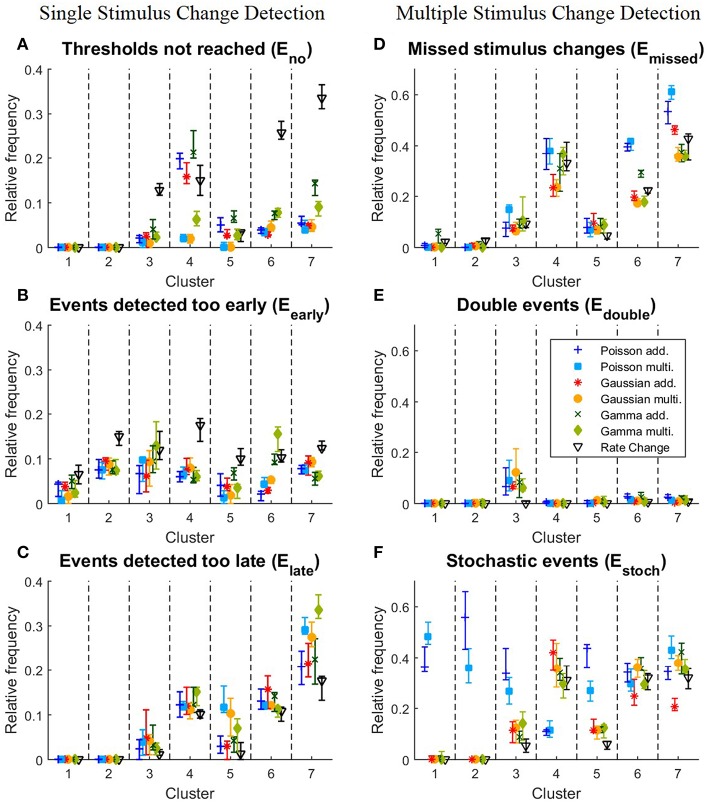
**Detailed analysis of the performances of the clusters for all 10 iterations of the stimulus protocol**. **(A–C)** Single stimulus change detection. **(A)** *E*_*no*_, relative frequencies at which the cumulative sum *S*_*t*_ did not reach any threshold for each cluster; **(B)** *E*_*early*_, relative frequencies for each cluster of events detected too early; **(C)** *E*_*late*_, relative frequencies for each cluster of events detected too late; **(D–F)** Multiple stimulus change detection. **(D)** *E*_*missed*_, relative frequencies for each cluster of missed stimulus changes; **(E)** *E*_*double*_, Relative frequencies for each cluster of stimulus changes detected twice; **(F)** *E*_*stoch*_, relative frequencies for each cluster of events detected under constant stimulation without changes. The relative frequency is defined as the number of corresponding events divided by the total number of stimulus changes in the cluster. The symbols show the median and the error bars illustrate the lower and upper quartiles.

We next examined, for the different response clusters, how well each of the methods detected events (Figure 4C) and which types of detection errors occurred for which model (Figures 5A–C). In Figure 4C the amount of correctly detected events (*E*_*true*_ in Figure 4B) was separated into the seven clusters of Figure 3. Most clusters with activity increases allowed a high rate of correct detections (clusters 1,2,3,5). For cluster 1, containing the most salient response increases, the multiplicative models yielded higher performances than the additive models and the Rate Change method. This observation can be explained by the higher rate of events detected too early by the additive CUSUM versions (Figure 5B). If an event was detected too early, the response to the real stimulus change was not investigated any more, because only one stimulus change per trace was assumed. For clusters 2, 3, and 5, all CUSUM methods yielded similar scores (80–95%) while the Rate Change method was less efficient. For the small but fast responses in cluster 4 the overall performances were lower in particular for the additive models and the Rate Change method, which yielded clearly lower detection scores than the multiplicative models (Figure 4C). Here, the decreased performance of the additive models can mostly be explained by the fact that in these cases of small response increases the cumulative sum did not reach the threshold, resulting in a high number of missed stimulus changes, *E*_*no*_ (Figure 5A). The two clusters containing activity decreases (6 and 7) yielded similar results for the Poisson and Gaussian but lower performance for the Gamma models. They tended to detect events too early in cluster 6 (Figure 5B) and not at all in cluster 7 (Figure 5A). For the first cluster with decreasing activity (cluster 6) the Poisson and Gaussian models detected over 75% of the stimulus changes (Figure 4C). As expected from the small changes in PSTH, the change point detection performances for the cluster 7 were generally lower than for the other clusters. Nevertheless, all methods were still able to detect over 50% of the velocity changes even in this difficult task. The additive models were found to yield a higher performance than the multiplicative models for detecting single changes, because they showed lower percentages of too late detections (Figure 5C). The Rate Change method detected activity decreases with a considerably lower performance than the CUSUM methods, because the threshold was often unreached (Figure 5A) or reached too early (Figure 5B).

The detection times of the correct events depended strongly on the previous and actual velocity, which is shown in Figure 4D. The detection time corresponded to the response latency and the activity differences (Figure 3B). For example, the fast and strong responses in cluster 1 allowed fast and accurate detection of stimulus changes with all versions of the CUSUM method. Sometimes detection occurred even before the latency adjusted time point of stimulus change (see Sections 2.1 and 2.3) leading to negative detection times. However, closer inspection revealed a trade-off between accuracy and speed of detection: While the multiplicative models detected more stimulus changes than the additives models, the multiplicative models tended to detect changes about 10 ms later than the additive models and the Rate Change, which required on average only 12 ms to detect a stimulus change in this cluster (Figure 4D). More generally, the multiplicative models tended to identify stimulus changes later than the additive models in clusters with only activity increases (clusters 1,2,4,5). Clusters with activity decreases (cluster 6,7) showed lower temporal accuracy, which is visible in the large interquartile range of detection times (Figure 4D). The very low median detection time of the models with low detection rates for activity decreases (in particular Gamma multiplicative and Rate Change) can be explained by a large percentage of random detections. The wide range of detection times observed for the biphasic responses in cluster 3 indicate that a change point was detected sometimes during the decreasing and sometimes during the increasing activity phase.

**Figure 6:**
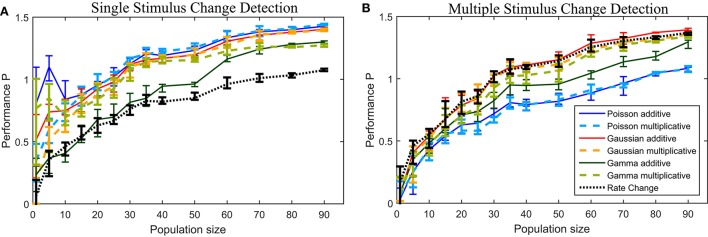
**Performance for down-sized data sets for the (A) single stimulus change detection and (B) multiple stimulus change detection**. See Section 2.6 for details on the down-sizing procedure and number of data sets used. The lines connects the median values and the error bars illustrate the lower and upper quartiles.

Figure 6A shows the performances for the down-sized data sets (see Section 2.6 for methods). The number of cells had qualitatively similar effects on the performances of all methods. For population sizes of about 30 cells or more the six CUSUM method showed stable results, except for the the Gamma additive model, which required a larger number of cells (~60). For some other methods (Poisson additive and Gamma multiplicative) the change point detection reached a very high performance for small numbers of cells (less than ten cells). However, the variability of detection performance was much higher for small populations than for larger cell numbers, suggesting that some individual cells might be more suitable for the change point detection task. As expected, the Rate Change method showed the poorest performance for all population sizes. In general, a smaller number of cells were required for the CUSUM method compared to the Rate Change method to reach a similar level of detection performance.

In summary, it was possible to detect changes for all types of response time courses with all six CUSUM methods. Both Poisson and Gaussian models achieved similar results, while the Gamma model showed a slightly lower total performance. The additive models did not detect small activity increases as precisely as the multiplicative models. On the other hand, the additive models tended to be slightly less impaired by false events, performed slightly superior for clusters with activity decreases and detected activity increases faster than the multiplicative models. The Rate Change method generally showed a lower performance. It could detect large activity differences, but it was often unable to detect activity decreases. Additionally it tended to detect events stochastically already before the actual stimulus change took place.

#### 3.2.2. Multiple stimulus changes

In this section the same data is analyzed as for the single stimulus change detection (Section 3.2.1) without preceding fragmentation. In Figure 2D an example of multiple stimulus change detection is illustrated with the additive Gaussian method. Here, the PSTH is smoother compared to the previous example (Figure 2C) because of the higher bandwidth Δ (see Table 4). Additionally both cumulative sums for the hypotheses of increased and decreased activity are shown in the time period of the analysis window (light gray region). In this example an activity increase with was detected about 20 ms after the stimulus change.

Figures 4E–H show the results of the multiple stimulus change point detection. The corresponding parameters are listed in Table 4B. In general, all seven methods allowed to detect a sequence of stimulus changes but showed clear differences in their performances. The Poisson models did not require a large bandwidth Δ, and used a shorter optimal reference window *R* (200 ms for additive, 300 ms for multiplicative), compared to the other models (400 ms). All other models required a longer bandwidth than the Poisson models using bandwidth of 1 ms (additive) or 10 ms (multiplicative). Both Gamma and the multiplicative Gaussian model had an optimal bandwidth of 20 ms, while the additive Gaussian and the Rate Change methods required an even longer bandwidth of 40 ms.

In Figure 4E the total performance *P* (Equation 7) is illustrated, and relative frequencies of true, missed and false events are shown in more details in Figure 4F (see Section 2.5.2 for definitions). Figure 4E shows that the Gaussian and Gamma models achieved similar performances as for the single stimulus case (Figure 4A). The frequencies of correctly detected events was on a similar level and in some cases even slightly higher when a sequence of change points had to be detected (Figure 4F). However, more false events occurred (ca. 25%) presumably due to the fact that the number of detected events was not limited like in the case of single stimulus detection. In contrast to the Gaussian and Gamma models, the Poisson models were significantly less efficient(~ − 30%) for multiple stimulus detection than for single stimulus change detection. Fewer correct events and more false events were detected. The reason for the poor performance of the Poisson models was that they detected a large number of stochastic events during constant stimulation. The Rate Change method yielded a much higher change point detection performance (~ +30%) compared to the case of single stimulus changes, with a similar performance as the Gaussian and Gamma CUSUM models.

The results for the different clusters are shown in Figure 4G. The performance variance within each cluster was often lower than for the single change case. As before (Figure 4C), the clusters 1,2,3,5 with peaks and increased steady state in population activity achieved high scores. In these clusters the detection rates were in fact increased compared to the single change detection task. Like in the case of single event detection cluster 4 yielded lower performances than the other clusters with activity increases. But in contrast to the single change detection additive and multiplicative models performed similarly, while the Poisson models clearly showed low performances. Detection rates of decreased activity in clusters 6 and 7 were significantly lower for the Poisson models than for the other models. Both Gaussian models and the multiplicative Gamma model achieved considerably high detection percentages for response decreases in cluster 6. They detected over 80% of these changes and the Rate Change method performed almost as well, while both Poisson models detected only approximately 60% of these stimulus changes. For cluster 7 the Poisson models performed particularly poorly. Only 40–50% of the stimulus changes were correctly detected with an approximately 10% lower detection rate compared to the single change case. Also the additive Gaussian model showed slightly lower performance than the remaining four models. The multiplicative Gaussian model performed similarly to the single change case, and only the Gamma models and the Rate Change method improved their performances.

To explain the observed differences between models for multiple stimulus detection, the distribution of the double (*E*_*double*_) and stochastic (*E*_*stoch*_) events (see Section 2.5.2) for the different clusters was analyzed (Figures 5E,F). All CUSUM methods had similar rates for detecting double events. These additional events were most often detected in cluster 3, containing responses to velocity changes from high speed in one direction to a high speed into the other direction (Figures 3A3,C3). Since the neurons responded with an activity dip followed by a peak, the methods first identified the reduced activity and after a short interval the increased activity. For stochastic events (Figure 5F) the Poisson models showed distinct properties compared to the other models. In the clusters 1, 2, 3, and 5, the Poisson models detected many more stochastic events than the other models. In contrast, the Poisson models detected only few stochastic events in cluster 4 where the other models identified stochastic events in 30–40% of the cases. The reason for these differences can be explained by the shorter optimal reference window *R* (see Table 4B and Discussion 4.4.1)

In Figure 4H the distributions of the correct detection times are shown. Despite similar distributions compared to the single stimulus change detection (Figure 4D), there were some differences: Here, no clear differences in detection times between multiplicative and additive models were found. While for the CUSUM methods detection times were about 5–10 ms longer in the case of multiple detections, the Rate Change method was considerably faster in the multiple detection case and even detected events in total about 5 ms earlier than the CUSUM models.

In Figure 6B the performance P for the down-sized data sets are shown. All methods, including the Rate Change method, showed a qualitatively similar dependency on the population size. Populations of at least 30–40 cells allowed reliable change point detection with the Poisson models requiring a larger population size and showing the lowest median performances in general.

In conclusion, it was possible for all seven models to detect multiple stimulus changes eliciting different response properties. The Gaussian, Gamma and Rate Change methods achieved similarly high performance scores, with the Rate Change method detecting the changes fast. In contrast, Poisson models more frequently missed velocity changes and detected stochastic events, resulting in poorer performances in particular for smaller populations of cells. Considering the results of both single and multiple change detection, the multiplicative Gaussian model yielded the highest and most consistent performances, but was not the fastest method.

## 4. Discussion

In this study, change point detection methods based on the CUSUM approach (Basseville, 1988) and a Rate Change method (Baker and Gerstein, 2001) were applied to neuronal data. Combining for the CUSUM methods three different assumptions on the underlying distribution of spike rates (Poisson, Gaussian and Gamma distributions) and two assumptions on the spike rate shift (additive and multiplicative), in total six different versions of the CUSUM approach (Table 3) were compared. We examined the performances of these six CUSUM methods and the Rate Change method for the detection of stimulus changes based on population responses of retinal ganglion cells to a moving light pattern. Changes in direction and/or speed of stimulus movement elicited characteristic neuronal responses. Depending on the type of a stimulus change, the population response either increased or decreased with different amplitudes and time courses. Therefore, the neuronal response was tested twice in parallel to identify change points of increasing and of decreasing activity, independently of each other (Table 2). Here, identical time dependent parameters (bandwidth of the PSTH and length of the reference window) were used for both hypotheses. However, nervous systems might employ different pathways for the two tasks of detecting activity increases and decreases. Even though the neuronal correlates of change point detection, e.g., by Bayesian neurons (Deneve, 2008), was not the scope of this study, we found that detecting activity decreases required a longer reference window and stronger smoothing of the PSTH. Nevertheless, assuming different parameter for both types of activity changes did not improve overall change point detection performance.

To bridge the gap between previous studies on neuronal change point detection (Ellaway, 1978; Churchward et al., 1997; Katz et al., 2001) and biological plausibility, the continuously recorded neuronal responses were analyzed in two different ways. In the traditional case of single stimulus change detection, the neuronal responses to each stimulus change were considered separately for a fixed period of time. In the biologically more realistic analysis of multiple stimulus change detection the neuronal responses were analyzed continuously without assuming prior knowledge on the number and timing of stimulus changes.

### 4.1. Single vs. multiple stimulus changes

Naively, one would expect the detection of multiple stimulus changes to be more difficult than the detection of individual stimulus changes. However, the Rate Change method, which is widely used in neuroscience, showed a lower performance for the single stimulus change detection than for the multiple case. This low performance could be due to the restriction of a maximum reference window length of 200 ms in the single case compared to 500 ms in the multiple case. However, even if the reference window length was doubled, the performance of single event detection did not significantly increase because the spike rate was not stationary. In principle, averaging in a moving reference window as applied to the multiple case could also be used for the single case. We also tested this method, but the performance with a moving reference window of 200 ms was even lower (results are not shown). Only if the reference window was increased to 400 ms, was the change point detection as efficient as for the CUSUM methods. For a reliable estimate of the mean and standard deviation, the moving average method seems to require a long reference window, which, however, cannot be achieved in the single change case with relatively short periods of stable conditions. For the CUSUM methods except for the Poisson methods, almost no difference in the number of detected stimulus changes was found between both tasks (Figures 4B,F). Separating the data into periods of constant length did not lead to an increased performance of stimulus change detections. On the other hand, the absolute performance was still higher for the single stimulus change case, because of lower relative frequencies of false events. These findings can be explained by the fact that the single change case allowed at most one false event (i.e., event detection ceased as soon as one event was detected), while in the multiple change case several events could be detected during the same stimulus response period.

The detection of multiple stimulus changes required stronger smoothing of the PSTH (i.e., larger PSTH bandwidth Δ; Table 4; see also Figures 2B,D for an example). The more strongly a PSTH is smoothed, the more symmetric the distribution of the data becomes. Therefore, Gaussian and Gamma assumptions are more compatible with activity distributions from smoothed PSTHs especially for PSTHs corresponding to stimuli eliciting low spike rates, which might account for the reduced performances of Poisson models for the multiple stimulus case.

Various strategies for detecting multiple change points have been discussed before. The most common strategy for a CUSUM method is the binary segmentation (Chen and Gupta, 2000). This offline analysis technique is based on cascaded change point detection. The data is split at the time of each change point and the periods before and after the change point are analyzed separately, until no additional change points are found. We did not adopt this approach, because we aimed for a method which relies exclusively on previous data. Additionally our method should be applicable to neuronal recordings of any length because in natural situations a change can occur at any time.

### 4.2. Additive vs. multiplicative

For the case of multiple stimulus change detection, virtually no differences between additive and multiplicative models were found. For the detection of single events corresponding to activity increases the multiplicative assumption yielded superior results, in particular for small increases. Although the multiplicative models showed higher performances for these clusters, they tended to detect the stimulus change later. Activity decreases, however, were detected to a slightly higher percentage by the additive models. This finding implies that different stimulus changes lead to non-consistent activity changes (multiplicative and additive) in the same data set. Some previous studies considered only additive shifts both in theoretical (Basseville, 1988) and neuroscience applications (Goense and Ratnam, 2003; Kim et al., 2012). In neuronal responses, however, multiplicative changes may also happen. In cortical neurons, for example, neural responses to varying inputs have been observed to change in a multiplicative way rather than additive (McAdams and Maunsell, 1999; Anderson et al., 2000; Sripati and Johnson, 2006; Wunderle et al., 2013). In such cases, a CUSUM method with an assumption of multiplicative changes may yield higher detection performances. Multiplicative changes of neuronal activity are also frequently discussed in the context of gain modulation, where the magnitude of one set of input (stimulus or synaptic) is related to the magnitude of another set of input (Salinas and Sejnowski, 2001; Chance et al., 2002). Gain modulations has been studied in single neurons, populations of neurons, as well as in network models. In many cases additive inputs have multiplicative effects on the output activity (Dayan and Abbott, 2005).

### 4.3. Distribution

The large number of different stimulus changes occurring in our data set led to a multitude of different activity distributions for the corresponding PSTHs. Hence, using an empirically determined distribution was not possible for this data set. Therefore, three alternative theoretical distributions (Poisson, Gaussian, and Gamma) were tested for their applicability, although statistical tests did not confirm that the data was distributed according to any of them. While the change point detection performances presented in the results section for the three underlying distribution assumptions speak for themselves, some additional theoretical aspects should be considered for the choice of the most appropriate CUSUM version for other data sets.

The Poisson distribution is a common assumption for modeling spike data, even though the Poisson hypothesis is sometimes rejected by experimental data (Amarasingham et al., 2006). However, it is often found in neuronal data that the expected value equals (approximately) the variance as it is the case for Poisson distributions. For the Gaussian and Gamma distributions the variance can also be set equal to the expected value. Alternatively, they require the fitting of one additional parameter, the variance σ^2^ for the Gaussian distribution and the shape parameter *k* for the Gamma distribution. We decided to fit these parameters and keep them constant during stimulus changes, as it was also done in other studies (Commenges and Seal, 1985). Another possible approach is to estimate these additional parameters continuously within the analysis window (Granjon, 2013), but this approach leads to a large fluctuation in the cumulative sum.

The Gaussian assumption has the fundamental theoretical disadvantage that it can never be fulfilled by PSTH data, which by definition cannot have negative values. Indeed, the PSTH data we used were not Gaussian distributed especialy when the average spike rates were low (Figures 1C–E). Despite this obviously violated assumption, both Gaussian models yielded high change point detection performances (Figures 4A,D), possibly suggesting that violations of underlying mathematical assumptions may not directly lead to low performances of the CUSUM method. Since the Rate Change method also implicitly assumes the Gaussian distribution, all of these theoretical arguments apply to it as well.

### 4.4. Parameters

#### 4.4.1. Reference window

In the CUSUM methods the reference window was used to estimate the previous mean (μ_0_) and if necessary the other parameters (σ^2^, *k*) of the assumed distribution. The Poisson models required shorter reference windows (see Table 4B) and behaved slightly differently from the other CUSUM versions for the multiple stimulus change detection. They detected a larger number of stochastic events especially in clusters with peaks of increased activity (see Figure 5F). To analyze this effect, we applied also a shorter reference window of 200 ms for both the Gaussian and Gamma models (see Table 4). The short reference window led to a higher probability of detecting stochastic events in classes with pronounced activity changes. In addition, a longer reference window (400 ms) was found to improve the detection of decreased activity.

For the results displayed in Figures 4–6 both cumulative sums for increased and decreased activities used the same PSTH and reference window. Our further analysis using different reference windows *R* and smoothing parameters Δ for the two different hypotheses revealed that testing for increased activity could be based on a shorter reference window and weaker smoothing of the PSTH. Activity decreases required a longer reference window as well as stronger smoothing of the PSTH. Applying a shorter reference window for detecting activity increases had a visible effect only on the detection performance of one cluster (no. 4), containing small activity increases, where the performance was poorer. Hence, detecting small activity differences generally requires a longer reference window than detecting big differences. As discussed in 4.1 the Rate Change method required a long reference window and stationary data to yield reliable results, leading to a much lower performance for single than for multiple event detection.

#### 4.4.2. Thresholds α_*in*_, α_*de*_, analysis window

The thresholds α_*in*_, α_*de*_ serve as the decision criterion whether the PSTH changed toward the hypothesis. For the CUSUM models the analysis window is the time period for the multiple stimulus change detection for calculating the cumulative sum *S*_*t*_. In theory, the thresholds α_*in*_, α_*de*_ depend on the so-called average run length (ARL). The ARL is defined as the expected number of samples before a change point is detected (Page, 1954; Basseville, 1988; Granjon, 2013). To determine an ARL, knowledge of the exact distribution of the data before and after the change is required (Hyu et al., 2010). Several approaches have been proposed to estimate the ARL, but they are computationally intensive or lead to poorer detection performances (Page, 1954; Siegmund, 1985; Granjon, 2013). In this study it was not possible to determine the ARL, because the changes in population activity had different time scales and different amplitudes. Therefore, the thresholds and the length of the analysis window were treated as regular parameters. The relative shifts (δ_*in*_, δ_*de*_) of the expected value were also strongly dependent on the length of the analysis window and the length of the reference window. Hence, it was not possible to optimize the parameters independently from each other, but they needed to be chosen in combination for the specific data set under study.

For the Rate Change method no analysis window was required. The thresholds α_*in*_, α_*de*_ for this method were multiples of the standard deviation. Generally, different threshold values for detecting activity increases and decreases were required, indicating that the activity did not vary symmetrically. The activity increases were much greater than the activity decreases. In the CUSUM methods, this asymmetry is reflected to the different values of the shifting parameter δ and its corresponding threshold α.

### 4.5. Application to other neuronal data

All seven models can in principle be applied to various types of neuronal data sets, although each model has its own advantages and disadvantages. In the same data set different assumptions can lead to similar performances in particular if different response dynamics are elicited by varying stimuli.

Neurons react to stimulus changes in various ways. In our data set taken from turtle retinal ganglion cells, we observed several types of transient and sustained response profiles of the pooled population (Figures 1B, 3B). Even biphasic responses and activity decreases were observed (Figure 3). The CUSUM methods were shown to be useful for change point detection based on these response profiles, which can be observed in similar shapes in many neuronal systems. The CUSUM methods are in general suitable for data sets that allow long integration times for the reference and analysis window. If the neuroscientific data set does not contain a sufficient number of spikes, the CUSUM methods compared in the study are not recommended. Instead, alternative methods that are based on individual spikes should be used, e.g., interspike interval analysis.

If the data set contains only one stimulus change, we suggest using a Poisson model with no or only little smoothing. The Poisson methods violated less theoretical assumptions than the other two distributions (see Section 4.3) and achieved similar performances as the Gaussian methods and slightly higher scores than the Gamma methods for single change detection (Figure 4A). If responses to multiple stimulus changes are present in the neuronal data, Gaussian and Gamma methods are preferable candidates. The Gamma methods may be slightly advantageous from a theoretical point of view, because the Gamma assumption is not explicitly violated (see Section 4.3). However, if the data set under study contains activity decreases to be detected, we recommend using Gaussian models (Figures 4C,G). If a long reference window with stable spike rates is available, alternatively the simple Rate Change method can be used for detection of multiple changes. However, the Rate Change method is not suitable for single event detection (Figure 4A).

Generally, some considerations about the specific data set need to be taken into account for the choice of a suitable change point method. Depending on the neural system under study, the range of latencies between stimulus and neuronal response may vary considerably. Thus, the borders of the reference window need to be defined accordingly. The time period between the starting point of the cumulative sum and the stimulus change should be in the same order as the interval accepted for the occurrence of correct events and in particular smaller than the minimal length of constant stimulation. The length of reference window should be determined under constant stimulation conditions. The relative shifts can be inferred from additive or multiplicative shifts between reference window and analysis window. After these considerations for suitable parameter ranges, it is advisable to test both additive and multiplicative versions of the CUSUM method for the specific neuronal response data set.

Here, we analyzed the spike rate as “mean” spiking activity of the cells. In principle, it is also possible to apply the method to other statistics of the spiking activity like Fano factor or variance. The Fano factor is often used in neuroscience as a measure for the variability in recorded spike trains especially when a Poisson process is assumed (Dayan and Abbott, 2005). Baker and Gerstein (2001) used the variance of the firing rate to detect latency jitters. These measures could be particularly useful to detect change points, when no obvious change in the PSTH is visible, because the cells react differently to stimulus changes or only few cells change their spiking behavior.

In summary, CUSUM methods provide a useful tool for the detection of stimulus changes based on changes in neuronal population activity. They are able to detect an unknown number of stimulus changes, which trigger both increases and decreases in activity. Hence, these methods are applicable to a wider range of neuronal data from different systems than the simple Rate Change approach. For our data set, high detection performances were achieved despite the fact that all assumptions were rejected by statistical tests. Hence, while violation of theoretical assumptions does not seem to impede the applicability of the CUSUM approach, the choice of model assumptions and parameters needs to be optimized for each specific data set under study.

## Author contributions

LK developed and implemented the methods, analyzed the results and wrote the paper. GA participated in data analysis and wrote the paper. JK participated in developing the methods, analyzed the results and wrote the paper.

## Funding

This work was supported by the FoL-Project (Forschungsorientierte Lehre) of the University of Oldenburg (LK) and by the DFG Cluster of Excellence EXC 1077/1 “Hearing4all” (GA, JK).

### Conflict of interest statement

The authors declare that the research was conducted in the absence of any commercial or financial relationships that could be construed as a potential conflict of interest.
