# Supplementary material for: Single and Multiple Change Point Detection in Spike Trains: Comparison of Different CUSUM Methods
Source: Front Syst Neurosci. 2016 Jun 22;10:51. doi: 10.3389/fnsys.2016.00051 (PMC4916211; doi:10.3389/fnsys.2016.00051)
Supplement: Supplementary file 1 [file Supplementarymaterial.pdf]

Supplementary Material:
Single and multiple change point detection in spike trains: Comparison of different CUSUM methods

Lena Koepcke, Go Ashida and Jutta Kretzberg*
*Correspondence:
Author Name: Jutta Kretzberg
jutta.kretzberg@uni-oldenburg.de

1 PERISTIMULUS TIME HISTOGRAM – PSTH

For constructing a smoothed PSTH y_t the spikes of all simultaneously recorded cells were pooled into a combined spike train. We did not pool over stimulus presentations but calculated a PSTH for every trial. For every time point t, y_t depends exclusively on the previous population activity. Let C be the number of cells and $\{x_1, \ldots, x_K\}$ (in ms) the spike times of all cells in one trial. We used two different smoothing methods, rectangular and 'half Gaussian' smoothing. A rectangular smoothed PSTH (spikes/sec) with a bandwidth Δ (in ms) is defined as

$$y_t = \frac{10^3}{C} \sum_{t-\Delta<x_i<t} \frac{1}{\Delta}. \tag{S1}$$

A 'half Gaussian' smoothed PSTH (spikes/sec) with a bandwidth Δ is defined as

$$y_t = \frac{10^3}{C} \sum_{x_i<t} \frac{\sqrt{2}}{\sqrt{\pi}\Delta} \exp\left(-\frac{(t-x_i)^2}{2\Delta^2}\right), \tag{S2}$$

Since both methods yielded similar results, only the results of the rectangular smoothed PSTH were shown in this article.

2 MATHEMATICAL DETAILS OF THE CUSUM METHODS

2.1 Poisson Process

In neuroscience, a spike train is often assumed to be a Poisson process, where the occurrence of a single spike depends solely on time and is independent of other spikes (see, e.g., **?**). The probability density function of a Poisson distribution is given by:

$$f(x,\mu) = \frac{\mu^x e^{-\mu}}{x!}, \qquad x \in \mathbb{N},\ \mu > 0. \tag{S3}$$

The expected value μ equals the variance. If a spike train is regarded as a Poisson process, this implies three mathematical assumptions.

1. There is at most one spike at one time point (a neuron cannot generate two spikes at the same time)
2. The spike counts in disjoint intervals are independent of each other.
3. The spike count y_t in an interval $[t, t+a]$ is Poisson distributed with the density function

$$f(y_t, \mu a) = \frac{(\mu a)^{y_t} e^{-\mu a}}{y_t!}, \tag{S4}$$

   with μ being the average spike rate.

The probability that k spikes occur in an interval $[t_i, t_i + a]$ of length a is the same for all t_i. We assume a Poisson process for the given spike train. Spike times are converted to an unsmoothed PSTH y_t with a bin size b. The value y_t is the number of spikes in $[t - b/2, t + b/2[$.

2.1.1 Additive shift of the expected value

Under the assumption of additive shifts, the general hypothesis of Eq. (1) (see Methods Section 2.2.1) is transformed into the following hypothesis.

$$H_0 : \mu_t = \mu_0 \; (\forall t) \quad \text{v.s.} \quad H_1 : \mu_t = \begin{cases} \mu_0, & t < c \\ \delta + \mu_0, & t \geq c \end{cases}, \qquad -\mu_0 < \delta < \infty, \tag{S5}$$

where μ_t is the spike rate at time point t and μ_0 the previous spike rate, which is calculated with the maximum likelihood estimator:

$$\mu_0 = \frac{1}{R} \sum_{i=t-R}^{t-1} y_i. \tag{S6}$$

R is the length of the reference window. The logarithmic residuals s_t from Eq. (2) (Methods Section 2.2.1 and Tables 1,3) are

$$\ln(f_{\mu_0+\delta}(y_t)) = \ln\left(\frac{(\delta+\mu_0)^{y_t} e^{-(\delta+\mu_0)}}{y_t!}\right) = y_t \ln(\delta+\mu_0) - (\delta+\mu_0) - \ln(y_t!), \tag{S7}$$

$$\ln(f_{\mu_0}(y_t)) = \ln\left(\frac{\mu_0^{y_t} e^{-\mu_0}}{y_t!}\right) = y_t \ln(\mu_0) - \mu_0 - \ln(y_t!), \tag{S8}$$

$$s_t = \ln(f_{\mu_0+\delta}(y_t)) - \ln(f_{\mu_0}(y_t)) = y_t \ln\left(\frac{\delta+\mu_0}{\mu_0}\right) - \delta. \tag{S9}$$

This leads to the recursive sum S_t.

$$S_0 = 0 \quad \text{and} \quad S_t = \max\left\{0, S_{t-1} + y_t \ln\left(\frac{\delta+\mu_0}{\mu_0}\right) - \delta\right\}. \tag{S10}$$

2.1.2 Multiplicative shift of the expected value

Under the assumption of multiplicative shifts, the general hypothesis of Eq. (1) (see Methods Section 2.2.1) is transformed into the following hypothesis.

$$H_0 : \mu_t = \mu_0 \quad (\forall t) \quad \text{v.s.} \quad H_1 : \mu_t = \begin{cases} \mu_0, & t < c \\ \delta\mu_0, & t \geq c \end{cases}, \quad \delta > 0,\ \delta \neq 1. \tag{S11}$$

μ_0 is calculated in the same way as before (Eq. (S6)). The log-likelihood in the time point t is calculated as:

$$\ln(f_{\mu_0\delta}(y_t)) = \ln\left(\frac{(\delta\mu_0)^{y_t} e^{-\delta\mu_0}}{y_t!}\right) = y_t \ln(\delta\mu_0) - \delta\mu_0 - \ln(y_t!), \tag{S12}$$

$$\ln(f_{\mu_0}(y_t)) = \ln\left(\frac{\mu_0^{y_t} e^{-\mu_0}}{y_t!}\right) = y_t \ln(\mu_0) - \mu_0 - \ln(y_t!). \tag{S13}$$

The recursive form of the CUSUM procedure (Eq. (2), Methods Section 2.2.1) can now be determined.

$$s_t = y_t \ln(\delta) + (1 - \delta)\mu_0, \tag{S14}$$

$$S_0 = 0 \quad \text{and} \quad S_t = \max\left\{0, S_{t-1} + y_t \ln(\delta) + (1 - \delta)\mu_0\right\}. \tag{S15}$$

2.2 Normal Distribution

With the assumption of normally distributed spike rates, the values of a PSTH at each time point $y_1, y_2, \ldots, y_n$ is assumed to obey the independent Gaussian distribution with variance σ

$$f_\mu(y_t) = \frac{1}{\sqrt{2\pi\sigma^2}} \exp\left(-\frac{(y_t - \mu)^2}{2\sigma^2}\right). \tag{S16}$$

The maximum likelihood estimators for μ and σ are the empirical mean and the empirical variance

$$\mu_0 = \frac{1}{R} \sum_{i=t-R}^{t-1} y_i, \quad \sigma^2 = \frac{1}{R-1} \sum_{i=t-R}^{t-1} (y_i - \mu_0)^2. \tag{S17}$$

2.2.1 Additive shift of the expected value

Under the assumption of additive shifts, the general hypothesis of Eq. (1) (see Methods Section 2.2.1) is transformed into the following hypothesis.

$$H_0 : \mu_t = \mu_0 \ (\forall t) \quad \text{v.s.} \quad H_1 : \mu_t = \begin{cases} \mu_0, & t < c \\ \mu_0 + \delta, & t \geq c \end{cases}, \quad -\mu_0 < \delta < \infty. \tag{S18}$$

The logarithmic residuals are

$$\begin{aligned}
\ln(f_{\mu_0+\delta}(y_t)) &= -\frac{1}{2}\ln\left(2\pi\sigma^2\right) - \frac{(y_t-(\mu_0+\delta))^2}{2\sigma^2}, && \text{(S19)}\\
\ln(f_{\mu_0}(y_t)) &= -\frac{1}{2}\ln\left(2\pi\sigma^2\right) - \frac{(y_t-\mu_0)^2}{2\sigma^2}, && \text{(S20)}\\
s_t &= -\frac{(y_t-(\mu_0+\delta))^2}{2\sigma^2} + \frac{(y_t-\mu_0)^2}{2\sigma^2}, && \text{(S21)}\\
&= \frac{\delta(2y_t-2\mu_0-\delta)}{2\sigma^2} = \frac{\delta}{\sigma^2}\left(y_t-\mu_0-\frac{\delta}{2}\right). && \text{(S22)}
\end{aligned}$$

This leads to the recursive sum S_t

$$S_0 = 0 \quad \text{and} \quad S_t = \max\left\{0, S_{t-1} + \frac{\delta}{\sigma^2}\left(y_t-\mu_0-\frac{\delta}{2}\right)\right\}. \tag{S23}$$

2.2.2 Multiplicative shift of the expected value

Under the assumption of multiplicative shifts, the general hypothesis of Eq. (1) (see Methods Section 2.2) is transformed into the following hypothesis.

$$H_0 : \mu_t = \mu_0 \ (\forall t) \quad \text{v.s.} \quad H_1 : \mu_t = \begin{cases} \mu_0, & t < c \\ \mu_0\delta, & t \geq c \end{cases}, \qquad \delta > 0. \tag{S24}$$

The logarithmic residuals s_t are:

$$\begin{aligned}
\ln(f_{\mu_0\delta}(y_t)) &= -\frac{1}{2}\ln\left(2\pi\sigma^2\right) - \frac{(y_t-(\mu_0\delta))^2}{2\sigma^2}, && \text{(S25)}\\
\ln(f_{\mu_0}(y_t)) &= -\frac{1}{2}\ln\left(2\pi\sigma^2\right) - \frac{(y_t-\mu_0)^2}{2\sigma^2}, && \text{(S26)}\\
s_t &= -\frac{(y_t-(\mu_0\delta))^2}{2\sigma^2} + \frac{(y_t-\mu_0)^2}{2\sigma^2} && \text{(S27)}\\
&= \frac{(1-\delta)\mu_0}{\sigma^2}\left(y_t-\frac{\mu_0(1+\delta)}{2}\right). && \text{(S28)}
\end{aligned}$$

The cumulative sum S_t was calculated by:

$$S_0 = 0 \quad \text{and} \quad S_t = \max\left\{0, S_{t-1} + \frac{(\delta-1)\mu_0}{\sigma^2}\left(y_t-\frac{\mu_0(\delta+1)}{2}\right)\right\}. \tag{S29}$$

2.3 Gamma Distribution

Under the assumption of the Gamma distribution, measured data points $y_1, y_2, \ldots, y_n$ are independently Gamma distributed with shape parameter k

$$f_\mu(y_t) = \frac{\left(\frac{k}{\mu}\right)^k}{\Gamma(k)} y_t^{k-1} \exp\left(-\frac{k}{\mu} y_t\right). \tag{S30}$$

The maximum likelihood estimator for μ is the empirical mean. Since a close form for the maximum likelihood estimator for k does not exist, an approximation form was used (**?**).

$$k^* = \ln\left(\frac{1}{R}\sum_{i=1}^{R} y_i\right) - \frac{1}{R}\sum_{i=1}^{R} \ln(y_i), \quad k \approx \frac{3 - k^* + \sqrt{(s-k^*)^2 + 24k^*}}{12s}. \tag{S31}$$

2.3.1 Additive shift of the expected value

Under the assumption of additive shifts, the general hypothesis of Eq. (1) (see Methods Section 2.2.1) is transformed into the following hypothesis.

$$H_0 : \mu_t = \mu_0 \ (\forall t) \quad \text{v.s.} \quad H_1 : \mu_t = \begin{cases} \mu_0, & t < c \\ \mu_0 + \delta, & t \geq c \end{cases}, \qquad -\mu_0 < \delta < \infty. \tag{S32}$$

The logarithmic residuals s_t are:

$$\ln(f_{\mu_0+\delta}(y_t)) = k(\ln(k) - \ln(\mu_0 + \delta)) - \ln(\Gamma(k)) + (k-1)\ln(x) - \frac{k}{\mu_0 + \delta} y_t, \tag{S33}$$

$$\ln(f_{\mu_0}(y_t)) = k\left(\ln(k) - \ln(\mu_0)\right) - \ln(\Gamma(k)) + (k-1)\ln(x) - \frac{k}{\mu_0} y_t, \tag{S34}$$

$$s_t = k\left(\ln(\mu_0) - \ln(\mu_0 + \delta) + y_t\left(\frac{1}{\mu_0} - \frac{1}{\mu_0 + \delta}\right)\right). \tag{S35}$$

The cumulative sum S_t was calculated by:

$$S_0 = 0 \quad \text{and} \quad S_t = \max\left\{0,\ S_{t-1} + k\left(\ln\left(\frac{\mu_0}{\mu_0 + \delta}\right) + y_t\left(\frac{1}{\mu_0} - \frac{1}{\mu_0 + \delta}\right)\right)\right\}. \tag{S36}$$

2.3.2 Multiplicative shift of the expected value

Under the assumption of multiplicative shifts, the general hypothesis of Eq. (1) (see Methods Section 2.2.1) is transformed into the following hypothesis.

$$H_0 : \mu_t = \mu_0 \quad (\forall t) \quad \text{v.s.} \quad H_1 : \mu_t = \begin{cases} \mu_0, & t < c \\ \mu_0 \delta, & t \geq c \end{cases}, \qquad \delta > 0. \tag{S37}$$

The logarithmic residuals s_t are:

$$\ln(f_{\mu_0\delta}(y_t)) = k(\ln(k) - \ln(\delta) - \ln(\mu_0)) - \ln(\Gamma(k)) + (k-1)\ln(x) - \frac{k}{\delta\mu_0}y_t, \tag{S38}$$

$$\ln(f_{\mu_0}(y_t)) = k\left(\ln(k) - \ln(\mu_0)\right) - \ln(\Gamma(k)) + (k-1)\ln(x) - \frac{k}{\mu_0}y_t, \tag{S39}$$

$$s_t = k\left(-\ln(\delta) + y_t\left(\frac{1}{\mu_0} - \frac{1}{\delta\mu_0}\right)\right). \tag{S40}$$

The cumulative sum S_t was calculated by:

$$S_0 = 0 \quad \text{and} \quad S_t = \max\left\{0,\ S_{t-1} + k\left(-\ln(\delta) + \frac{y_t}{\mu_0}\left(1 - \frac{1}{\delta}\right)\right)\right\}. \tag{S41}$$

3 PARAMETER OPTIMIZATION

3.1 Optimization of the thresholds α

For each tested parameter combination, the thresholds α_{in} and α_{de} were computed to maximize the value of P (see Eq. (6) in Methods Section 2.4). Initial values were set for α_{in} and α_{de}, and then the values were shifted upwards and downwards first with a big step size (e.g., a step size of 10) until the best performance was reached. This procedure was performed repeatedly with shrinking step sizes until the best performance was reached by a step size of 0.25. The optimization was done for each of the 10 iteration of the stimulus protocol independently.

3.2 Optimization of the parameter combinations

The basic idea of optimizing the parameter combinations was to go from coarse to fine. First a coarse grid of the parameter combinations was tested. Then every parameter of the best combinations was varied repeatedly with a finer grid until the best performance was achieved. For the single case four parameters have to be optimize (bandwidth Δ, relative shifts δ_{in}, δ_{de} and length of reference window R). For example, the initial set of tested bandwidths was $\{1, 5, 10, 30, 50, 70\}$ and the reference window R $\{50, 100, 150, 200\}$ ms. If, e.g., the bandwidth of 30 ms and the length of 150 achieved a good result, for the next set of tested bandwidths was $\{20, 25, 30, 35, 40\}$ combined with reference windows $\{100, 125, 150, 175, 200\}$. Often, several parameter combinations achieved good results. Therefore, all parameter combinations, which had a performance $P > 1.3$ or were within the 20, were further investigated.
